# Synergistic Interventions for Silencing Oppositional Defiant Disorder and Dyslexia in a Child With Attention-Deficit/Hyperactivity Disorder: A Case Report From Albania

**DOI:** 10.7759/cureus.61753

**Published:** 2024-06-05

**Authors:** Rigels Kurushi, Mohamed Omer, Omer Hussein, Majid Ali, Anas Ibn Auf

**Affiliations:** 1 Medicine, Sulaiman Alrajhi University, Al Bukayriyah, SAU; 2 Executive Department, ADHD Hyperactivity Albania Foundation, Tirana, ALB; 3 Psychiatry, Prince Sultan Military Medical City, Riyadh, SAU; 4 Psychiatry, Erada and Mental Health Complex, Taif, SAU; 5 Psychiatry, Eastern Sudan College for Medical Sciences and Technology, Port Sudan, SDN

**Keywords:** albania, pcit, odd, adhd, heavy metal detoxification, parent-child interaction therapy, dyslexia, oppositional defiant disorder, attention deficit hyperactivity disorder

## Abstract

To the best of the authors’ knowledge, this article is the first of its kind in Albania and neighboring countries to investigate the transformative synergistic intervention approach through cognitive behavioral therapy, parent-child interaction therapy (PCIT), and heavy metal detoxification on a child with attention-deficit/hyperactivity disorder (ADHD) and comorbid oppositional defiant disorder (ODD) and dyslexia. The limited mental health awareness in Albania, particularly regarding PCIT and similar treatments, highlights the importance of the applicability and adaptability of such interventions. This study suggests that the rapid management of comorbidities in ADHD, such as ODD and dyslexia, is better achieved by a combined intervention approach and by investigating the biological aspects. Further research with a large sample size is needed to assess the long-term sustainability and scalability of such an approach.

## Introduction

Attention-deficit/hyperactivity disorder (ADHD) is a neurodevelopmental disorder characterized by an ongoing pattern of inattention and/or hyperactivity-impulsivity that interferes with functioning or development. It is accordingly classified into three subtypes: predominantly inattentive, predominantly hyperactive-impulsive, and combined [1], often pervasive across different fields. Academic, social, and personal areas are disrupted, with learning and social interactions also hindered, resulting in a notable decline in the daily functioning of those affected. The impact of ADHD seen in various aspects of life points to the complex and difficult nature of this neurodevelopmental disorder [2]. The impact of ADHD is not only seen in the academic lives of children, even though it is the most important. ADHD affects their social interactions and self-esteem significantly [3]. Everything becomes a daily struggle, from creating new friendships to controlling impulses or recognizing social cues, something that later on brings isolation, loneliness, and even depression. Such a mechanism will lead to a lower self-image and confidence, which is fueled by the constant “war” of coping with everyday stressors and self-regulation due to a heightened emotional response and regulation [4].

ADHD is very common in childhood [5], and it tends to present with comorbidities. Oppositional defiant disorder (ODD) is one of the most common comorbidities of ADHD and is characterized by defiant, hostile behavior, especially toward authority figures [6]. It is common for children, especially those two to three years old, to be oppositional and defiant, but in ODD, this behavior is manifested frequently through frequent temper tantrums, argumentativeness with adults, vindictiveness, and a refusal to comply with rules or requests [7]. This becomes a persistent pattern, and the behaviors become challenging. This will later significantly impact the child’s social interaction and family life, causing a struggle to form healthy relationships [8]. ODD and ADHD are thought to share some behavioral traits and etiological factors. This complex comorbidity increases the difficulty of a tailored treatment, especially for ADHD. Poor communication and hostility toward treatment, together with oppositional behavior [5], make ODD a difficult obstacle for the treatment regime of ADHD.

One of the therapies used to address disruptive behaviors in children, as seen in ODD, is parent-child interaction therapy (PCIT) [9]. The main goal of this intervention is to emphasize positive parent-child interaction since many parents find it very difficult to deal with their children’s behaviors, often leading to parents’ anxiety and depression [10]. The treatment typically consists of two phases: the child-directed interaction (CDI) and the parent-directed interaction (PDI) [9,10]. Each phase starts with a teaching session followed by coaching sessions in which parents are coached in vivo by the therapist while interacting with their child. In CDI, parents learn to follow their child’s lead [9], helping create a medium for addressing behavioral challenges by teaching the parents how to effectively encourage compliance and positive social interaction.

Another common comorbidity of ADHD is a specific learning disorder with impairment in reading, according to DSM5, or dyslexia as it is referred to in educational contexts [11]. This is a learning disorder manifested by difficulties in reading, spelling, and decoding words. Neurobiological investigations of dyslexia have shown low neural activity in the left temporoparietal and occipitotemporal regions, brain regions responsible for language processing [12]. This leads to a wider scope than just reading difficulties; spelling, writing, and comprehension skills are also affected. This highlights the need for a tailored intervention that is multisensorial in nature. Structured literacy programs, assistive technologies, and personalized educational support are some of the approaches to it, aiming to nurture neuroplasticity [13] and facilitate adaptive neural pathways in order to enhance reading proficiency, thereby helping individuals achieve a better understanding of their surroundings.

Beyond genetics and behavioral studies, there are observations of the impact of heavy metals such as lead, mercury, chromium, and arsenic on neurodevelopment [14]. Exposure to such substances may contribute to synaptic disruption, cognitive deficits, and impairment of the neurotransmission system [15], leading to an exacerbation of behavioral difficulties, as seen in ADHD and ODD.

This case study, presented with the consent of the family, shows a very unique approach to a rare case, exploring some interconnected subjects while offering an integrated approach for broader management, focusing not only on the child but also the parent and environment, and highlighting the importance of a multifaceted intervention when dealing with comorbidities. Such an approach helps break down complex cases through objective reasoning, setting a strong foundation for a holistic approach to pediatric care.

## Case presentation

We present the case of a seven-year-old Albanian male diagnosed with ADHD at three years of age, exhibiting a complex clinical profile. He had a congenital absence of the right forearm and hand and a comorbid ODD diagnosed at six years old. The child took lisdexamfetamine (50 mg in the morning) for his ADHD symptoms and had been receiving cognitive behavioral therapy (CBT) [16] for seven months. A few weeks after commencing the CBT, the child was found to have dyslexia. His mother reported that, beyond the clinical presentation, lay a difficult family dynamic. The mother was taking citalopram for her depression, and the strained relations with her husband, the child’s biological father, added another layer of complexity. The child had no siblings, and neither parent had any long-term organic diseases. However, the intense conflict between the mother and father had significantly affected the family dynamics, to the point where they had even discussed divorce. As a result, the home environment for the child had become increasingly difficult. The child was expelled from his first day of primary school due to his disruptive behavior. His mother reported that her primary concerns were his aggressive outbursts, verbal hostility, and disruptive conduct, as he constantly fought with peers during his time at school and displayed verbal aggression toward teachers.

Assessment and interventions

Knowing the congenital limb malformation, together with the geographical proximity of the child’s residence to a city in Albania where there are multiple chromium mines, led to the hypothesis of elevated heavy metal levels. This hypothesis was supported by many past reports of such cases in that area, as well as past family history reported by the mother; thus, the first step was to order a urinalysis via inductively coupled plasma mass spectrometry (ICP-MS) [17] at a certified private laboratory. With regard to the psychological manifestations of both the child and his mother, five different assessment tools were used in order to reduce the measurement bias as much as possible. The Eyberg Child Behavior Inventory (ECBI) [9] was utilized as a home evaluation to assess the frequency and severity of disruptive behaviors. Another measure, the Dyadic Parent-Child Interaction Coding System (DPICS) [9], was utilized for the mother-child interactions during PCIT. An important approach was implemented with the help and consent of the child’s new school, in which a specialized education teacher assessed his disruptive behaviors in a school setting. During the child’s cognitive-behavioral therapy sessions at the ADHD Albania clinic, the Wide Range Achievement Test, Fifth Edition (WRAT-5) [18] was conducted to assess dyslexia. This instrument is used to capture any potential developmental shifts, especially those related to comorbidities. For that reason, the measures were separated into three distinct phases: the preparatory phase, the exploratory phase, and the graduation phase. The measures were repeated every four months.

Preparatory Phase

**Figure 1:**
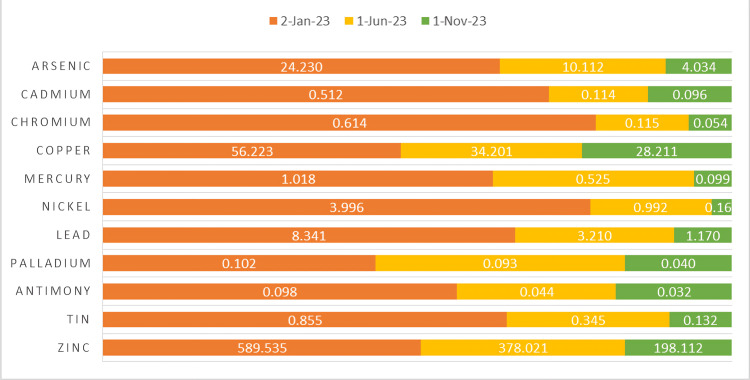
ICP-MS The reference lab values of metal in urine are as follows: arsenic <25.0 μg/L, cadmium <0.50 μg/L, chromium <0.4 μg/L, copper <2.0-80.0 μg/L, mercury <2.3 μg/L, nickel <3.30 μg/L, lead <4.5 μg/L, palladium <0.4 µg/L, antimony <0.25 μg/L, tin <1.8 µg/L, and zinc <85.0-1,250.0 μg/L. ICP-MS, inductively coupled plasma mass spectrometry

Since the child was living near the chromium mines, the primary focus of the initial phase of this investigation was the assessment of the levels of heavy metals in the child. In order to do that, a small panel of urine heavy metals was ordered using ICP-MS in a German-certified private laboratory in the capital city of Tirana, Albania. The values were normalized using creatinine levels. As expected, the analysis revealed elevated levels of several metals, notably nickel and lead, with arsenic and cadmium at the higher boundaries of the normal range. The other metals in the small panel urinalysis - copper, mercury, palladium, antimony, tin, and zinc - were within an acceptable range, even though they were at the high end of the spectrum (Figure 1).

After the results of the urinalysis, a consultation with the child’s pediatrician followed. Based on a full checkup, consideration for chelation therapy was ruled out, with the rationale that it had been reserved for only acute cases. Instead, a tailored dietary regimen was recommended. The dietician prepared a regimen incorporating foods and drinks rich in compounds such as N-acetylcysteine and alpha-lipoic acid, as well as some natural chelators such as curcumin found in high levels in turmeric, Atlantic Dulse, spirulina, and chlorella extract supplements. The diet was to be followed three times a day, seven days a week, together with light morning exercise.

**Figure 2:**
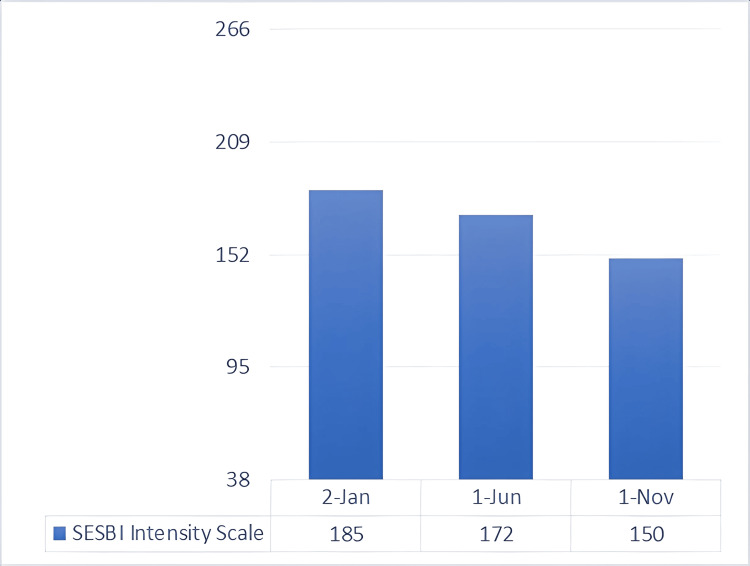
SESBI intensity scale: baseline and post-intervention scores This is a rating scale used to measure the frequency of disruptive behaviors in children (minimum score = 38, maximum score = 266, and raw score cutoff for clinical significance: ≥151). SESBI, Sutter-Eyberg Student Behavior Inventory

**Figure 3:**
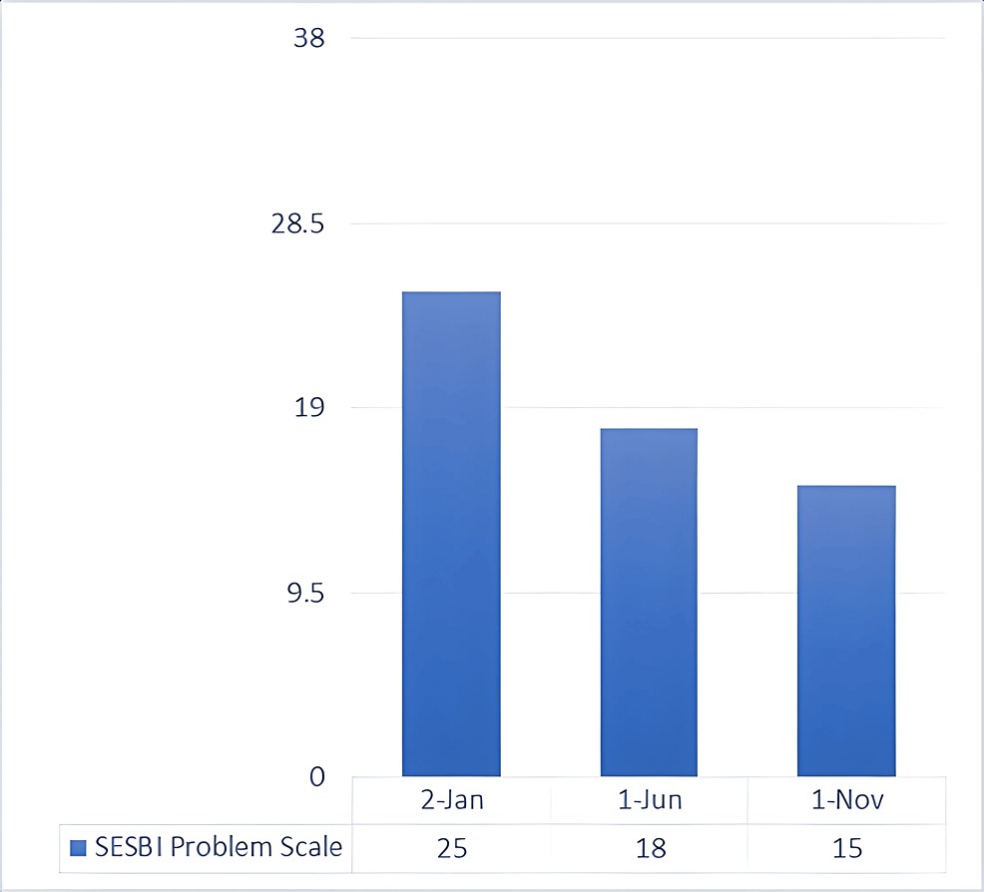
SESBI problem scale: baseline and post-intervention scores This is a rating scale used to measure the degree of disruptive behavior in children (minimum score = 0, maximum score = 38, and raw score cutoff for clinical significance = ≥19). SESBI, Sutter-Eyberg Student Behavior Inventory

**Figure 4:**
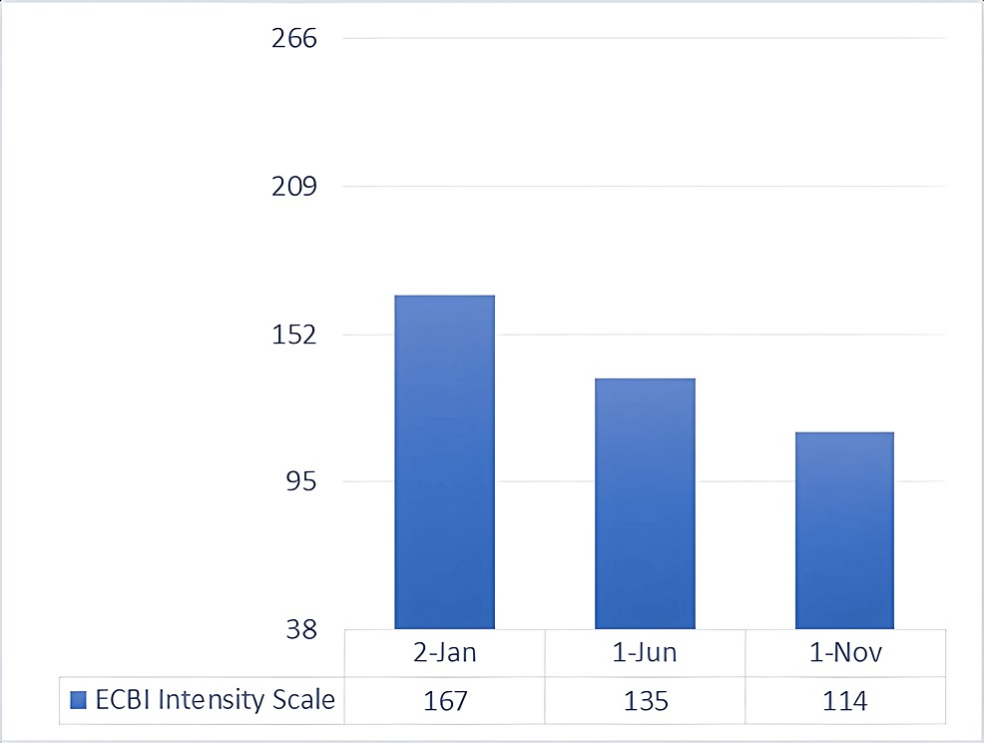
ECBI intensity scale: baseline and post-intervention scores This is a rating scale used by parents to rate the intensity of disruptive behaviors in children (minimum score = 36, maximum score = 252, and raw score cutoff for clinical significance = ≥127). ECBI, Eyberg Child Behavior Inventory

**Figure 5:**
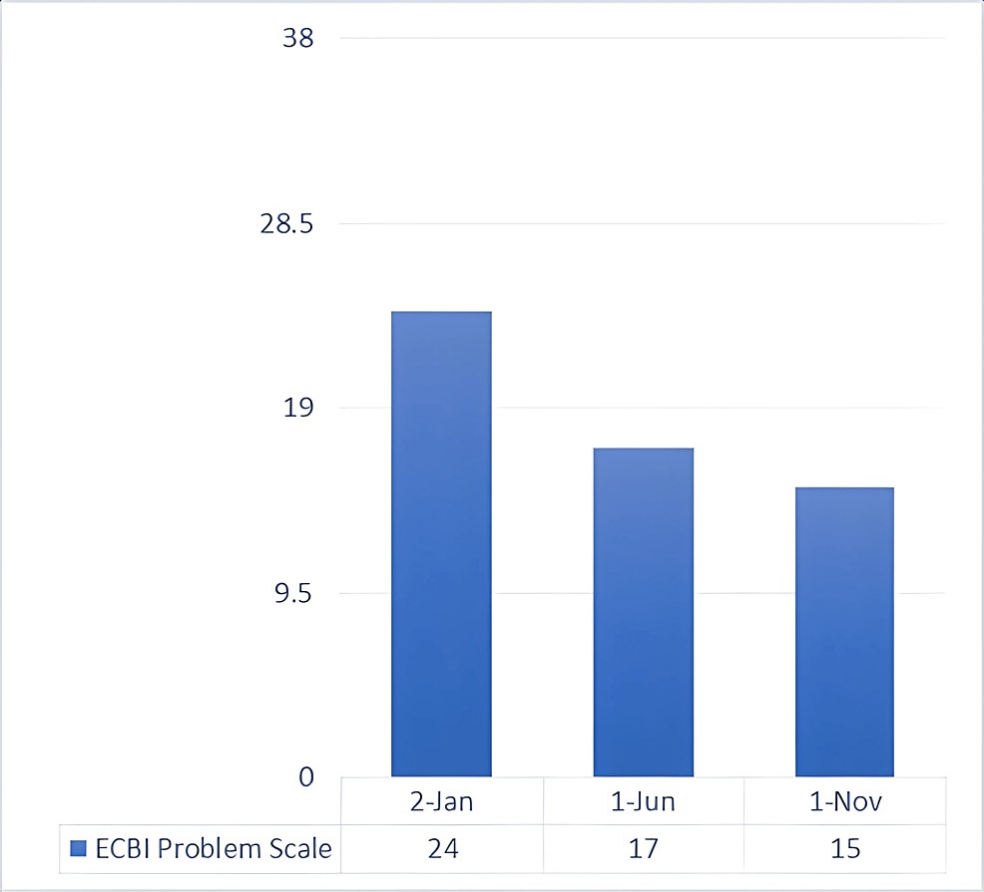
ECBI problem scale: baseline and post-intervention scores This is a rating scale used by parents to indicate whether each behavior is a problem for them (minimum score = 0, maximum score = 36, and raw score cutoff for clinical significance = ≥15). ECBI, Eyberg Child Behavior Inventory

Since the mother’s main complaint when visiting the clinic was the behavioral disruption culminating in his expulsion from school, the next area of focus was the facilitation of readmission to either the same or another school. Following a meeting with the school faculty, which included members of the treatment team, he was readmitted, and a special education teacher was assigned to him. With her consent, she would take part in the evaluation of the child’s treatment. The teacher was a Sutter-Eyberg Student Behavior Inventory (SESBI) [19] and ECBI master; thus, together with the mother of the child, an assessment plan was founded. The scales are divided into two components: the intensity scale and the problem scale. The first approach was the evaluation from the special education teacher, with the help of the SESBI scale (Figures 2, 3), a validated questionnaire designed to be completed by teachers and healthcare providers, which registered a value of 185 for the SESBI Intensity Scale (baseline 151) and 25 for the Problem Scale (baseline 19). In order to have a greater reduction of measurement bias, the ECBI scale, a validated questionnaire designed to be completed by parents, was used by the mother in order to make a better comparison (Figures 4, 5). The assessment revealed an intensity scale of 167 (baseline 117) and a problem scale of 24 (baseline 15).

**Figure 6:**
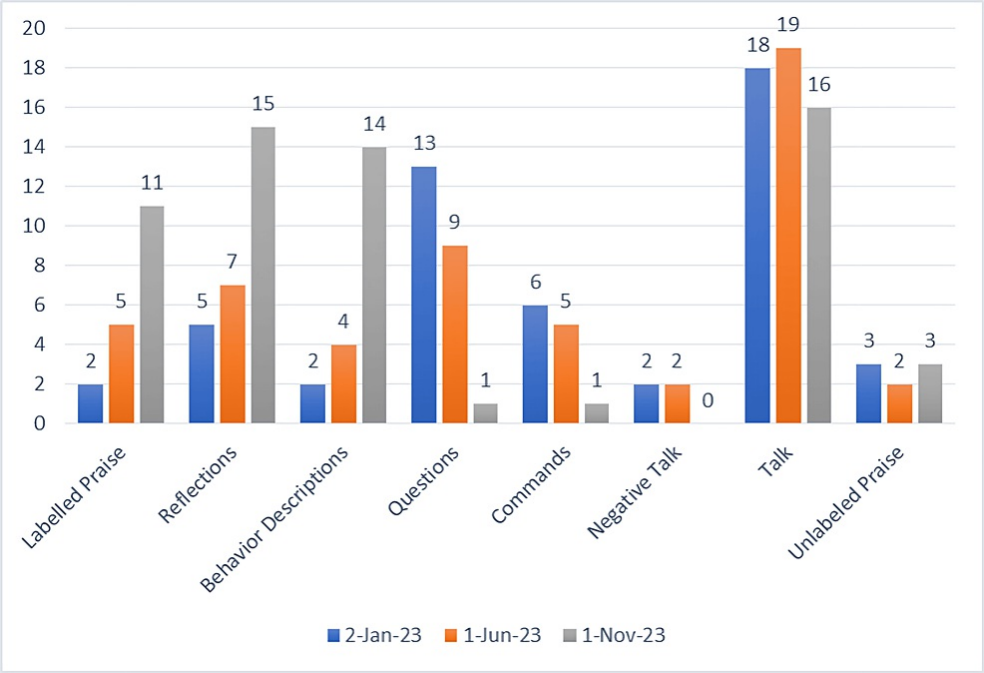
Results of DPICS baseline and post-intervention scores for parent-child interaction This is a clinician-observational measure of the caregiver’s parent-child interaction behaviors. The observed behaviors are summarized as three “do” skills: praise, reflect, and describe (goal of ≥10 for each skill), and three “don’t” behaviors: questions, commands, and criticisms/negative talk (goal of ≤3 when all three are combined). In addition, two behaviors are considered neutral (neither “do” or “don’t” skills), including talk and unlabeled praise. DPICS, Dyadic Parent-Child Interaction Coding System

The next step of the preparatory phase was addressing the mother’s perspective. She was introduced to PCIT and its modality. The mother underwent a brief introduction regarding PDI and CDI skills, as well as the Praise, Reflect, Imitate, Describe, and Enjoy (PRIDE) approach [9] within the framework of PCIT. During three days of teaching sessions regarding the framework and the reach of PCIT, the mother was given directions on the DPICS, which she was expected to complete at the end of the introductory week. The scale consists of three “DO” skills, with a baseline value of 10 each, and three “DON’T” skills, with a targeted score of ≤3 for all three skills, as well as two neutral skills. The results of the test (Figure 6) necessitated an augmented therapeutic intervention consisting of four sessions per week.

**Figure 7:**
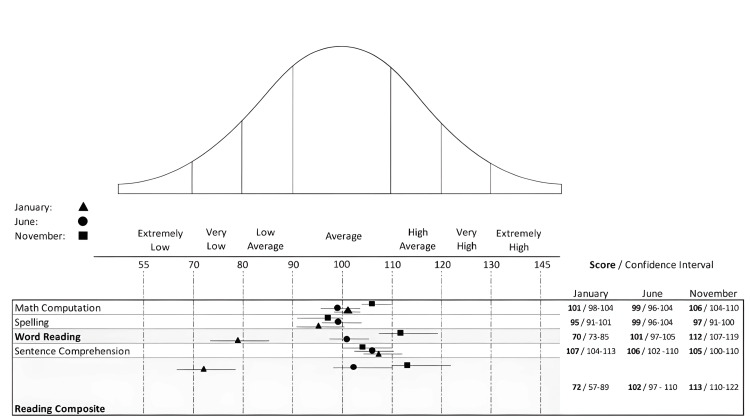
Results of the WRAT-5 for the different phases of the study WRAT-5, Wide Range Achievement Test, Fifth Edition

After a tailored approach to the most concerning presenting complaints, we shifted the focus toward the most recent finding, the child’s dyslexia. In response to that, our objective was to develop a standardized approach to addressing this condition. The WRAT-5, together with the Wechsler Intelligence Scale for Children, Fifth Edition (WISC-V) [18], were used to objectively assess the presence of a specific learning disorder, in that case, dyslexia, as well as evaluate the treatment efficacy. After a series of tests, the clearest evidence was in the Ability-Achievement Discrepancy Analysis [20] (Table 1), in which the spelling, word reading, and reading composite were disrupted, with the spelling on the lower spectrum. The Pattern of Strengths and Weaknesses Analysis (Table 2) supported the theory of a specific learning disability (SLD), namely dyslexia, as clinically evident during the CBT sessions. The combined reports are standardized and put in a bell curve based on the monthly reports for each phase of this study (Figure 7).

**Table 1:** Ability-Achievement Discrepancy Analysis: WRAT-5 predicted vs. actual scores The predicted WRAT-5 score refers to the score expected based on the individual’s overall ability level, typically derived from cognitive assessments or other relevant measures. The WRAT-5 score indicates the actual performance achieved on the WRAT-5. The scores of each one of the subtests in the table should range between 55 and 145. WRAT-5, Wide Range Achievement Test, Fifth Edition

WRAT-5 subtest/composite	Predicted WRAT-5 score	WRAT-5 score	Difference	Significance level	Base rate
Math computation	92	101	-	-	-
Spelling	93	85	8	<0.01>	≤15%
Word reading	92	70	22	<0.01>	≤10%
Sentence comprehension	92	107	-	-	-
Reading composite	92	72	20	<0.01>	≤10%

**Table 2:** Pattern of Strengths and Weaknesses Analysis: significant differences supporting the SLD hypothesis The relative strength score indicates the performance level in areas where the individual demonstrates stronger abilities compared to their overall cognitive profile. The relative weakness score indicates the performance level in areas where the individual demonstrates weaker abilities relative to their overall cognitive profile. The scores of each one of the subtests in the table should range between 55 and 145. SLD, specific learning disability

Comparison	Relative strength score	Relative weakness score	Difference	Critical value (0.01)	Significant difference (Y/N)	Supports the SLD hypothesis (yes/no)
Processing strength/achievement weakness	89	75	14	14	Y	Yes
Processing strength/processing weakness	89	68	21	15	Y	Yes

The CBT sessions for the child were continued, even though slightly modified to suit a more tailored approach regarding his behavioral symptoms and SLD derived from the tests and clinical diagnosis. A detailed plan was made for both the child and the mother five times a week until the next phase, with the aim of achieving a “graduation phase” as soon as possible where all the parameters fell within their respective ranges.

Exploratory Phase

Toward the end of the preparatory phase, there was already positive feedback from the developmental therapist, special teacher, and mother regarding the child’s behavioral symptoms. Thus, the synergistic approach needed to be evaluated for its efficacity. As expected, there was already a decrease in both the intensity and problem scales of SESBI (Figures 2, 3), evaluated five months after the initial phase of testing. The same went for the ECBI scale (Figures 4, 5), even though it is still above the borderline. Continuous feedback from the CBT sessions regarding the child’s dyslexia was confirmed by WRAT-5 analysis, where a huge jump was seen in word reading and reading composite, already within the average range of the curve. It seemed like the child never had that disorder, yet it was too early to graduate and have a clear conclusion. As for the other subtests (math computation, spelling, and sentence comprehension), minor changes were observed, all within the average range (Figure 7).

Capitalizing on these positive developments, nearly no changes were made to the therapeutic regime, including the detox diet, for which another ICP-MS urinalysis was sent to the lab. Remarkably, within a mere four months into the detox diet, every value fell within the acceptable range (Figure 1), even though, again, it was too early for a clear conclusion. The results were interpreted by the child’s pediatrician and dietician, who encouraged the mother to keep using the same therapeutic regime for the child, as well as some physical exercise. As for the child’s medications, lisdexamfetamine was still continued at the same dose. Also, citalopram for the mother remained unaltered, followed by a consultation with the family psychiatrist.

Particularly noteworthy was the improvement of the mother-child bond. When first observed in the clinics, the child had multiple tantrums, and the mother had difficulties responding to his behavior. In the second phase of this study, the mother had a completely different approach toward her child, something that was clearly reflected in the child’s response to not only CBT but also in the school setting. DPICS was done again, and the results were promising, even though not as expected (Figure 6).

Graduation Phase

During the exploratory phase, extending from mid-June to the end of October, the therapeutic plan was strictly followed by both the mother and child sides. Slight but not significant changes were made to the diet, along with the introduction of morning jogging and light exercise by the mother. In addition to that, a strategic decision was taken: changing the residence from his city (near chromium mines) to Tirana, the capital city of Albania, after the graduation phase, for further reduction of heavy metal levels in the child. To our surprise, the graduation phase came earlier than expected, with some remarkable results from mother and child, which motivated this case study to be developed.

The last urinalysis results (Figure 1) from ICP-MS were shared with the family physician, who acknowledged the accomplishment by ruling out any pharmacological chelating therapy for the child and further making evident the efficacy of the detox diet in this child’s case. Together with a multisystemic checkup, the conclusion to stop the official diet regimen was given, even though the mother was eager to continue it indefinitely with some slight modifications.

Multiple collective feedback, given by different teachers in the child’s school, was altogether reflected in the SESBI evaluated by the special education teacher, with an intensity scale of 150 and a problem score of 15, both meeting the criteria for graduation (Figures 2, 3). Similar results were seen in the ECBI scale reported by the mother, registering an intensity score of 114 and a problem score of 15, further validating the graduation and the evidence of not meeting the criteria for ODD and all behavioral symptoms (Figures 4, 5).

Another surprising finding came from the mother, who, just one week before the graduation phase, stopped using citalopram under the guidance of her psychiatrist. There was a remarkable improvement in the mother-child bond, leading to some unexpected results in DPICS, where the “DO” skills scored more than 10 each and the “DON’T” skills altogether scored only 2, further clarifying the graduation phase and ensuring all the criteria were met (Figure 6). The child’s tantrums seemed to have disappeared, as continuous feedback was given to the mother, developmental therapist, and multidisciplinary team.

The most remarkable and unexpected discovery was the child’s dyslexia, which showed complete resolution. During the CBT sessions, there were multiple subjective tests given to the child with words including letters B, R, S, and C in which the child initially exhibited significant difficulties. However, at the graduation phase, the child not only achieved proficiency in writing these words but also in reading words that, before the preparatory phase, he could not read at all. The multiple accounts were reflected in the remarkable improvement in the word reading and reading composite domains of WRAT-5, scoring 112 (CI = 107-119) and 113 (CI = 110-122), respectively. This transitioned from a very low spectrum in the preparatory phase to average in the exploratory phase, and now to high average in the graduation phase (Figure 7). The ability achievement and the pattern of strengths and weaknesses failed to notice any SLD, further validating the complete resolution of dyslexia.

## Discussion

Heavy metal distribution and accompanying pollution are rather extensive, necessitating prompt action in some cases. Limiting cleanup to a single method would be ineffective and unsafe for heavy metal removal. Furthermore, the disadvantages and limitations of one method of remediation can be overcome by combinatorial approaches. Thus, a multidisciplinary approach using multiple physicochemical and biological strategies is necessary to combat heavy metal pollution [9].

Heavy metals are naturally occurring elements with relatively high atomic weights and a minimum density of five times that of water. They have numerous applications in businesses, households, agriculture, and medicine, which contributes to their widespread distribution in the environment. Most heavy metals are extremely poisonous. They have a variety of exposure pathways, including ingestion, inhalation, and skin absorption, leading to various health impacts from human-heavy metal contact. Heavy metals have been shown to have more severe effects on children's health than on adults. The negative effects on children’s health include mental retardation, neurocognitive disorders, and behavioral disorders [8].

This case study documents a remarkable transformation observed in a seven-year-old child who was suffering from numerous behavioral problems. These issues significantly impacted his schooling and had the potential to severely affect his later life if left untreated. The careful and thorough evaluation and management have led to a complete resolution of the previously diagnosed ODD stemming from his behavioral symptoms. Additionally, a newly discovered SLD, identified as dyslexia, has been addressed. This transformation further strengthened the mother-child bond, which was fragile in the early phases of this study but showed continuous positive development by the end of the research.

A synergistic, customized therapeutic approach contributed significantly to this transformation, particularly following the confirmation of the “high heavy metal theory” established during the case presentation. This approach encompassed a detox diet, CBT, and PCIT. This innovative combined approach was introduced for the first time in Albania due to limited awareness of such interventions. Our ongoing research aims to raise awareness for the adoption of healthier approaches to mental disorders and to broaden therapeutic interventions to encompass not only the individual but also their environment.

PCIT was found to be an effective intervention for child disruptive behavior disorders, but the small number of eligible studies and lack of variety in the sample populations indicate the need for additional studies. This study has significant importance for both practitioners and researchers and provides a concise synopsis of the research to date [11].

Overall, existing findings indicate reasonably robust associations for ADHD with negative urgency, positive urgency, lack of premeditation, and lack of perseverance. Additionally, there is reasonable support for associations between ODD and negative urgency. More importantly, these findings suggest the need to reexplore and revise the trait impulsivity hypothesis in terms of the unique associations of different dimensions of impulsivity with the externalizing symptoms of ADHD and ODD. However, existing literature that addresses such a comprehensive evaluation is limited [3].

It is important to mention the constraints of our study, which have the typical limitations of any case study design, such as the absence of a control group and limited generalizability. Primarily, the impact of each component - CBT, PCIT, and the detox diet - needs closer inspection, as does the pharmacotherapy, even though the dosage did not change and the CBT sessions continued as before. Secondly, a longer time frame is needed to draw definite conclusions, as well as follow-up sessions.

A key restriction is the statistical approach used in previous investigations. Although there is significant overlap in variances across the externalizing spectrum of symptoms and impulsive characteristics, this was not adjusted for in the statistical studies. As a result, multicollinearity may confound the findings. It would be beneficial to account for such confounding effects [3].

## Conclusions

To the best of the authors’ knowledge, this case report is the first of its kind in Albania and neighboring countries, making evident the transformative journey of a child and his mother by standard measures. It also underscores the importance of a holistic approach, prioritizing the symptoms over the diagnostic labels themselves. Further follow-up and study replication are needed to extend these findings. Future studies with randomized controlled trials are recommended to ensure the consistency of these findings. Follow-up investigations will help determine how long the therapeutic effects last once the treatment session is completed.
